# Patient demographics, clinical characteristics and genetic mutations of DMD and BMD patients in Qatar Epidemiological and genetic profile of Duchenne muscular dystrophy and Becker muscular dystrophy patients in Qatar: a retrospective cohort study

**DOI:** 10.3389/fped.2025.1569505

**Published:** 2025-08-01

**Authors:** Mohammad Sawahreh, Fatima Al-Maadid, Khalid Omer Ibrahim, Tawfeg Ben Omran, Mahmoud Fawzi Osman

**Affiliations:** ^1^ Department of Pediatrics, Division of Pediatric Neurology, Sidra Medicine, Doha, Qatar; ^2^ Department of Pediatrics, Division of Genetic and Genomic Medicine, Sidra Medicine, Doha, Qatar

**Keywords:** Duchenne muscular dystrophy, Becker muscular dystrophy, dystrophinopathy, mutation analysis, Qatar, cognitive impairment, gene therapy

## Abstract

**Background:**

Duchenne muscular dystrophy (DMD) and Becker muscular dystrophy (BMD) are rare X-linked neuromuscular disorders that typically begin in childhood and progress to functional decline, loss of ambulation, and early death due to cardiac or respiratory failure.

**Objective:**

To describe the landscape of DMD and BMD in Qatar, including demographics, genetics, disease progression, risk factors, co-morbidities, and outcomes in patients aged 3–30 years, and compare findings with international data.

**Methods:**

We retrospectively reviewed records of all genetically confirmed or biopsy-supported cases of DMD and BMD between 2018 and 2024 at Sidra Medicine, the sole pediatric tertiary center in Qatar.

**Results:**

Of the 37 symptomatic patients (36 DMD, 1 BMD), 36 were male and one was a symptomatic female. The mean age was 18 years (range 3–30). At diagnosis, median age was 3.0 years. Twenty-two (59%) had orthopedic complications (scoliosis, contractures), 9 (24%) could still run, and 12 (32%) could climb stairs. Corticosteroids were prescribed in 14 patients (38%), most commonly deflazacort and prednisone. Cardiac medications were started in 7 patients (19%) around age 10. CK was elevated in 36/37 (range: 2,300–45,000 U/L). Epilepsy was documented in 3 patients; 3 had autism and 1 had ADHD. Genetic mutations included deletions (69%), duplications (11%), and point mutations (19%). Seven patients had mutations affecting Dp140/Dp71 isoforms and cognitive impairment.

**Conclusions:**

Our cohort reveals earlier diagnosis but lower life expectancy compared to international standards, likely due to lower corticosteroid and cardioprotective use. The findings support the need for strengthened multidisciplinary and early genetic-based interventions in Qatar

## Introduction

Duchenne muscular dystrophy (DMD) and Becker muscular dystrophy (BMD) are rare, progressive, and X-linked recessive neuromuscular disorders caused by mutations in the Dystrophin gene (*DMD*) in the Xp21 region (1). DMD is more common affecting 1 in 5,000 live male births, and typically presents with early motor symptoms such as difficulty climbing stairs and waddling gait appear by ages of 2 and 3 years (2). Progressive muscle degeneration in corticosteroid-naïve boys often results in loss of ambulation by the age of 10 and 12, and assisted ventilation by the age of 20 (2).

Cardiac and/or respiratory failure between the ages of 20 and 40 is the leading cause of death amongst DMD patients (3). Muscle weakness leads to clinical manifestations such as Trendelenburg gait, calf pseudohypertrophy, lumbar lordosis, and scoliosis (4). BMD on the other hand, is a milder form with a slower progression, a later age of onset and longer life expectancy (2).

At the molecular level, Thousands of mutations in *DMD* have been found in patients with DMD. Major predominant types are deletions (∼60%), duplications (∼10%) and point/splice/small mutations (∼30%) (5). These mutations disrupt dystrophin protein isoforms such as Dp427m (muscle), Dp140 and Dp71 (brain) (5) affecting muscular and neurodevelopmental function (6). Additionally, Neurological manifestations may include intellectual impairment (defined as intelligence quotient <70), learning difficulties, autism, behavioral and psychiatric disorders, epilepsy and attention-deficit hyperactivity disorder (ADHD), particularly when mutations disrupt Dp140/Dp71 expression (7).

In the case of BMD, a shortened less functional form of dystrophin is produced leading to the less severe form of the disease, that's because unlike DMD, in BMD the mutations retain the reading frame (5).

Diagnosis typically involves elevated creatine kinase level (could reach levels >20,000 U/L in DMD patients due to damage to the plasma membrane of muscle cells), genetic testing via Multiplex Ligation-dependent Probe Amplification (MLPA) or Next Generation Sequencing (NGS) and in rare cases, diagnostic muscle biopsy (8). In accordance with current worldwide standards of care, physical therapy and corticosteroid therapy are approved to alleviate symptoms and slow disease progression after diagnosis is confirmed (4).

Interestingly, recently published literature unveiled that in the Middle East and North Africa (MENA) the awareness of the disease is low and clinical approaches were all inconsistent to the international guideline and recommendations published in 2018 (9). Therefore, in this article we are trying to provide insights on the demographics, clinical characteristics, genetic mutations and management protocols available currently in Qatar. This will help improving the care of children affected with these conditions in many ways:

- **Clinical Management**: Better diagnostic strategies, treatment decisions and provision of appropriate supportive care tailored to the specific needs of patients in Qatar.
- **Genetic Counseling**: Accurate epidemiological and genetic data are essential for providing genetic counseling to affected families. Understanding the prevalence and inheritance patterns of DMD and BMD in Qatar allows healthcare providers to offer informed guidance regarding the likelihood of recurrence in future pregnancies and the implications for other family members.
- **Efficient Resource Allocation**: This includes planning for diagnostic services, specific therapeutic interventions, and support programs for affected individuals and their families based on the anticipated prevalence and needs of the population.
- **Research Work**: By elucidating the unique genetic landscape of DMD and BMD in Qatar, researchers can contribute to advancements in precision medicine and targeted therapies that may benefit patients in the region and beyond.
- **Public Health Initiatives**: Understanding the epidemiological profile of DMD and BMD facilitates the development and implementation of public health initiatives aimed at raising awareness, promoting early detection, and improving access to healthcare services for affected individuals. This can lead to earlier diagnosis, better management of symptoms, and improved quality of life for patients with DMD and BMD in Qatar.

## Methods

### Data collection

This retrospective cohort study was conducted at Sidra Medicine, Qatar. We reviewed medical records from January 2018–February 2024 for patients with a diagnosis of DMD or BMD confirmed by genetic testing or muscle biopsy.

**Inclusion criteria:**

- Clinical features consistent with DMD or BMD
- Genetic confirmation by MLPA, NGS, or other sequencing techniques
- Or muscle biopsy showing absent/reduced dystrophin staining (anti-Dys1/Dys2)

**Data collected:**

- Demographics: nationality, age, sex, consanguinity
- Clinical features: motor delays, stair climbing, running ability, scoliosis/contractures
- Laboratory: CK, ALT, AST (range and elevated proportion)
- Comorbidities: epilepsy, autism, ADHD, statural delay
- Treatment: corticosteroids (type, age at initiation), cardiac medications
- Genetic mutations and inheritance patterns

Ethical approval was obtained. Data were anonymized.

## Results

At the date of this study, there are 37 records of patients with genetically confirmed DMD and BMD patients and carriers from all over Qatar; of these, 6 were patients that died between the ages of 14 and 20 due to respiratory failure, heart failure or complications due to their scoliosis corrective surgeries. Out of the two females that carried a dystrophinopathic gene variant, one was excluded from further discussions in this paper as she was only an asymptomatic carrier, but her dizygotic twin brother was included.

### Demographics and clinical presentation

DMD Patient demographics and their clinically significant characteristics are summarized in Table 1. Out of the 37 patients (36 DMD, 1 BMD). The mean age was 18 years (range: 3–30). Fifteen were Qatari and the rest were expatriates from India (7), Egypt (4), Yemen (2), Pakistan (2), with the remaining are single patients from Syria, Poland, Afghanistan, Kuwait, Iraq, Lebanon and Sudan. Twenty-five were from unrelated families. Consanguinity was reported in 12 families. Median age at diagnosis was 3.0 years (range: 1 month to 13 years). Twenty-two patients (59%) presented orthopedic complications that include scoliosis, lordosis and various contractures in the lower limbs. Nine (24%) could run; twelve (32%) could climb stairs. Twenty patients had gross motor delays, 9 patients had speech delays. Cognitive impairment was seen in 11 patients (30%), including three patients with autism, seven patients with intellectual disability and a single patient with ADHD. Three patients (8%) had epilepsy. Statural delay was seen in four corticosteroid-treated patients.

**Table 1:** Summary of patient demographics, clinical characteristics and laboratory findings.

Patient characteristics	Number of patients (percentage of total)
Demographics, *n* (%)	
Males	36 (97)
Females	1 (3)
Alive	31 (84)
Dead	6 (16)
Qatari (local)	15 (40)
Expatriates	22 (59)
Consanguinity, *n* (%)	
Yes	12 (32)
No	25 (68)
Diagnosis, *n* (%)	
DMD	36 (97)
BMD	1 (3)
Age at first diagnosis, years	
Mean (SD)	3.8 (3)
Median (min, max)	3 (0.03, 13)
Learning difficulties, *n* (%)	
Yes	11 (30)
No	21 (57)
Data not available	5 (13)
Intellectual impairment, *n* (%)	
Yes	7 (19)
No	20 (54)
Data not available	10 (27)
Full-time wheelchair user, *n* (%)	
Yes	16 (43)
No	20 (54)
Data not available	1 (3)
Patient able to run, *n* (%)	
Yes	9 (24)
No	25 (67)
Data not available	3 (8)
Patient able to climb stairs, *n* (%)	
Yes	12 (32)
No	22 (59)
Data not available	3 (8.)
Corticosteroid treatment, *n* (%)	
Yes	14 (38)
No	21 (57)
Data not available	1 (3)
Patients with abnormal laboratory results, *n* (%)	
Elevated CK	36 (97)
Elevated CK (>20,000 U/L)^a^	6 (17)
Elevated ALT	28 (76)
Elevated AST	27 (73)

^a^ Proportion of patients with abnormal CK levels that exceeded 20,000 U/L.

Patients with clinically abnormal laboratory assessment results for ALT (range: 70–250 U/L), AST (range: 60–210 U/L), and CK (range: 2,300–45,000 U/L) levels, as compared with reference ranges, were 75% (28/37), 73% (27/37), and 97% (36/37), respectively.

### Management plans

Corticosteroids (38%) are the most reported choice for initial management along with medications prescribed for other diseases secondary to DMD. The specific corticosteroids used were Deflazacort in 10 patients and Prednisolone in 4 patients initiated at a median age of 6 years (range: 4–10); treatment regimens varied in dose and schedule (daily vs. intermittent) based on physician discretion and family preference. The BMD patient was followed for monitoring but did not require corticosteroid or cardiac treatment at the time of data collection, as his symptoms were mild. All are summarized in Table 2.

**Table 2:** Summary of reported management plan.

Management plan	Number of patients (percentage of total)
Medication, *n* (%)	19 (51)
Physical Therapy, *n* (%)	37 (100)
Devices, *n* (%)	16 (43)
Surgery, *n* (%)	4 (11)
Other^a^, *n* (%)	3 (8)
Medications, *n* (%)	
Corticosteroids	14 (38)
Cardiovascular diseases agents	7 (19)
Devices, *n* (%)	
Wheelchair	17 (46)
Respiratory Aids	8 (22)

^a^ Patients opted for stem-cell based and homeopathic therapies outside of Qatar, i.e., China and India.

Cardiac involvement was seen in ten patients (27%), with onset between ages 9–15. Seven patients (19%) were started on ACE inhibitors or β-blockers, typically at age 10. Three patients tried stem-cell therapy abroad at age 9–11; all are non-ambulatory.

The most commonly used device for supportive management are wheelchairs (46%) then respiratory aids (BiPAPs and Ventilators) at 22%. Three patients reported to have tried either Stem-cell based therapy or homeopathic therapy outside of Qatar with no observable benefit, as all are currently wheelchair bound.

It must be noted that the BMD patient was followed for monitoring but did not require corticosteroid or cardiac treatment at the time of data collection, as his symptoms were mild.

### Genetic analysis

A summary of the different types of genetic mutations that were recorded for all the patients are shown in Table 3. Mutations were detected in 36/37 only, as the analysis of two patients was not conclusive so they were excluded, but it should be noted that Anti-Dys1 (rod domain) and Anti-Dys2 (carboxy-terminal domain) antibodies were used in both cases, showing absent dystrophin expression consistent with a diagnosis of DMD. Out of the thirty-six, ten patients fell into four families; while the remainder of the patients are not known to be related to each other. Deletions accounted for 69% of all mutations, in which twenty-three were diagnosed with DMD, one with BMD and one that was a DMD carrier. Four DMD patients had duplication type mutation and the remaining had small mutations. Five of the patients diagnosed with DMD had a *de novo* mutation.

**Table 3:** Number of patients with different mutations.

Mutation Class	DMD	BMD
Deletion	23	1
Duplication	4	-
Point	6	-
Splice	1	-
Undetermined^a^	2	-
***De novo***	5	-

^a^ Absent dystrophin expression.

### Special cases

Interestingly, there was a symptomatic DMD female with a *de novo* deletion mutation presented at age 7 with difficulty walking, had CK of 28,000 U/L, and muscle biopsy showed absent dystrophin expression using anti-Dys1 and abti-Dys2 antibodies. One DMD patient having a co-occurring LAMA2 pathogenic variant presented at age 3 years with hypotonia, delayed milestones, currently non-ambulatory with brain MRI revealing periventricular white matter changes and muscle biopsy showing partial dystrophin and complete merosin deficiency. The BMD patient (30 years old) was ambulatory with mild symptoms, no cardiac and didn't receive any steroid treatment.

## Discussion

This is the first and largest study to be published of DMD and BMD patients in Qatar to date which provides insights into demographics, clinical characteristics, and genetic data from thirty-seven patients diagnosed to have DMD or BMD. As shown in Table 1. 40% of the patients are local while the rest are expatriates. The lower local to expatriate ratio is aligned with the overall social construct of Qatar, in which according to the latest census by the Planning and Statistics Authority (2020), the local population is 11% only of the overall population. The percentages in the study are more than what is provided in the census, which is due to possible familial clustering given there are six Qatari patients with same deletion that are siblings leading to a higher prevalence, additionally due to the limited size of the data set, minor changes in the number of participants led to bigger changes in the percentage.

The mean age at diagnosis was 3.8 years which is within the normal age range of DMD symptoms onset of approximately 2–3 years of age (4). This number is much lower than what was reported in a study from Saudi Arabia (6.9 years) and in an expert opinion about the situation in the MENA region that reported 7.8 years (11, 12). The numbers are also aligned with the numbers reported in the CARE-NMD project and the Strategic Targeting of Registries and International Database of Excellence (STRIDE) (13). In our study, we compared the average age at death among our DMD patients only (excluding the single BMD patient), with the DMD cohort reported by Broomfield et al. (14), who included only patients with genetically confirmed Duchenne muscular dystrophy. The average age was slightly lower than the reported mean age of 22 for patients born before the 1990s and 29.9 for those born after the 1990s (14) with some reports even showing patients reaching their 40s (15). According to the data (Table 1), the average age of patients with DMD that already died was 18 (14, 23), but as currently the standard cardiopulmonary support is being followed, we expect the average life expectancy to increase with the remainder of the patients (4). The lower average age at death (mean 18 years) in our DMD cohort, compared to the 22–29.9 years reported by Broomfield et al., may be attributed to differences in treatment coverage. In our cohort, only 38% of DMD patients were on corticosteroids, and 19% received cardiac medications. In contrast to the Broomfield study, which reported corticosteroid use in over 90% of patients and near-universal use of ACE inhibitors and β-blockers by age 10. The reduced access or uptake of these therapies in our population has likely contributed to the shorter life expectancy observed.

In accordance with current worldwide standards of care, management includes physical therapy, corticosteroids, and cardioprotective agents such as ACE inhibitors and β-blockers, which are associated with delayed progression of cardiomyopathy and improved cardiac outcomes should be initiated as early as possible before the significant decline in physical ability to increase patient life expectancy (4), that is why all patients underwent physical therapy as part of the standard DMD care plan in Qatar, but unfortunately, only 38% of patients are reported to be on corticosteroids when compared to the 86% reported in the Cooperative International Neuromuscular Research Group Duchenne Natural History Study, and 89% in the STRIDE Registry (13, 16). Despite the difference in numbers, the low use of corticosteroids was not surprising, as comparable numbers have been reported in neighboring countries such as in Saudi Arabia with 40.7% of patients only receiving corticosteroids (12) In further review of records, few families refused to consent for the use of corticosteroid-based treatment, which could be due to the potential side effects (9, 17).

The loss of ambulation is an important indicator of disease progression due to extensive muscular dystrophy, thus tracking at what age the patients became wheelchair bound may indicate the effectiveness of disease management plans implemented or severity of the underlying mutation (18). The records unveil that 46% of patients diagnosed with DMD became wheelchair bound or need a wheelchair to assist with long distances. The mean age is 9.6 years of age, which is consistent with previous reports that came out of the Middle East (9, 12, 19) but still lower than the reported 13 years of age in UK North Star database and other worldwide studies (20, 21).

**Figure 1:**
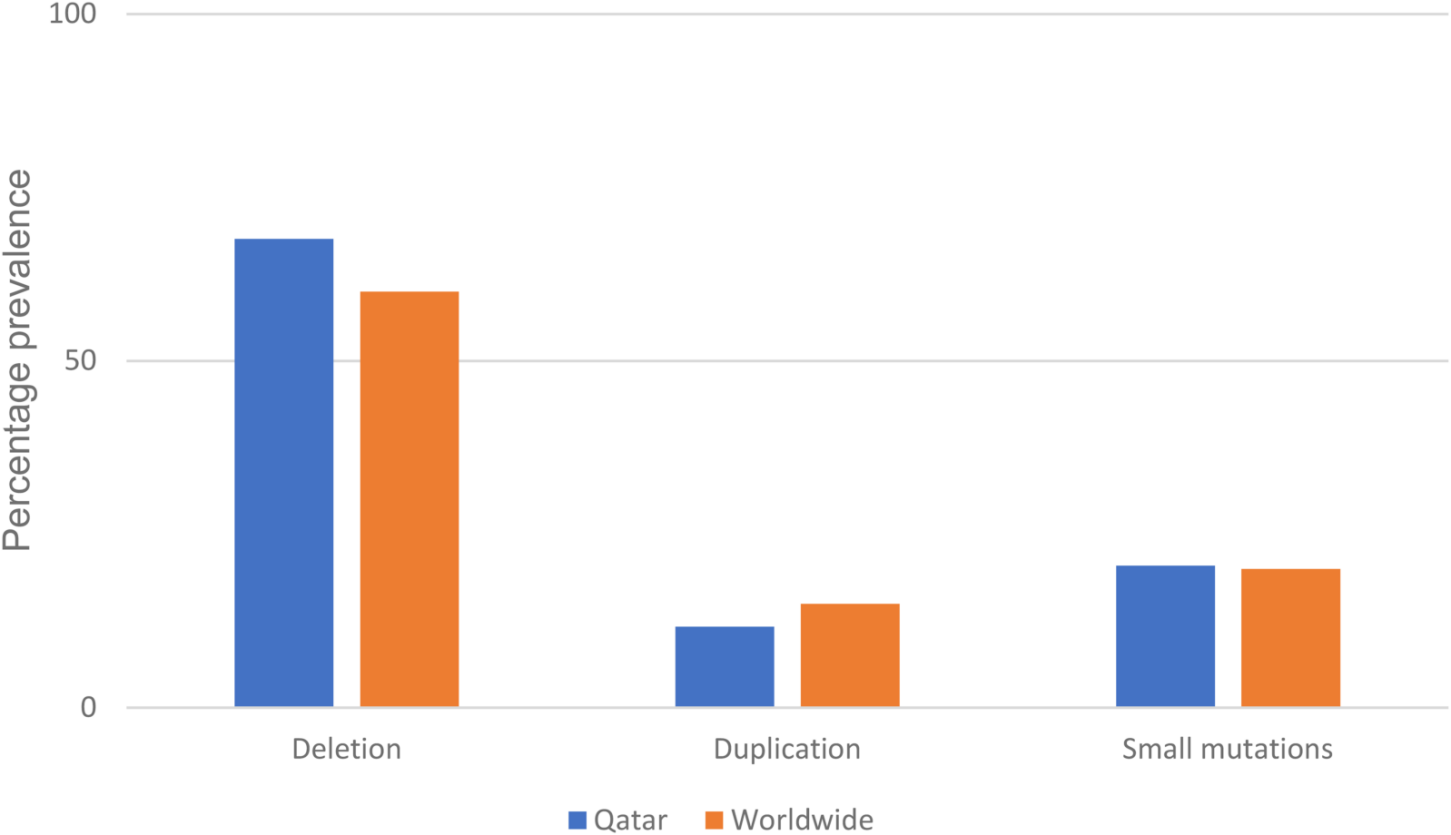
Percentage prevalence of different DMD mutations recorded compared to world-wide reported prevalence (10).

Finally, to find a correlation between the type and position of mutations with place of origin as a tool for future quicker treatment options for patients in the region, we summarized all mutations of diagnosed patients in Table 4. Deletions account for nearly 69% of all mutations in this data set. This number is aligned with real world reported data of 60% (Figure 1) (5). Going deeper into the data, 54% of the reported deletion mutations were in the reported hotspots of exons 44–55 and 3–9, with the prevalence of 47% and 17% respectively, which is also in alignment with previously reported data (22) Duplications account for 11% of all patient mutations, with 50% of the mutations occurring in the previously reported hotspots of 3–11 and 21–37 (23). Unfortunately, assumptions regarding reading frames cannot be made solely based on genomic testing, and further mRNA analysis should be made to establish the orientation of the duplicated fragment. 19% of reported mutations were small mutations, and as expected, the mutations are not concentrated at a certain hotspot and are evenly distributed. We also have identified previously reported point mutations, the c.7657C > T(p.R2553X) and c.3151C > T(Arg1051) (24).

**Table 4:** Genetic data of all DMD/BMD patients enrolled in this study.

Number of Patients	Mutation Class	Position	Diagnosis	Nationality	Comments
6	Deletion	Exon 8–34	DMD	Qatari	Siblings and Cousins (one affected female)
3	Deletion	Exon 45, 46, 47	DMD	Qatari	*De Novo*
1	Deletion	Exon 45–52	DMD	Egyptian	-
1	Deletion	Exon 52, 53, 54	DMD	Qatari	-
1	Deletion	Exon 49, 50	DMD	Indian	-
2	Deletion	Exon 12–25	DMD	Indian	Siblings
1	Deletion	Exon 51	DMD	Yemeni	-
1	Deletion	Exon 3–13	DMD	Qatari	-
1	Deletion	Exon 48, 49, 50	DMD	Egyptian	-
1	Deletion	Exon 45–54	DMD	Indian	-
2	Deletion	Exon 48–79	DMD	Pakistani	Siblings.
1	Deletion	Exon 55	DMD	Qatari	*De Novo—*Female
1	Deletion	Exon 3	BMD	Qatari	-
1	Deletion	Exon 60, 61, 62	DMD	Qatari	-
1	Deletion	Exon 10–17	DMD	Kuwaiti	-
1	Duplication	Exon 61, 62	DMD	Qatari	*De Novo*
1	Duplication	Exon 63–79	DMD	Iraqi	-
1	Duplication	Exon 18	DMD	Sudani	-
1	Duplication	Exon 2–16	DMD	Polish	-
1	Point/nonsense	Exon 22	DMD	Syrian	Pathogenic variant
1	Point	c.2378A > G (p.N793S)	DMD	Qatari	*De Novo—*Likely Pathogenic mutation
1	Point Stop	Exon 35	DMD	Egyptian	LAMA2 pathogenic variant also detected
1	Splice	Intron, IVS21 + 1G > A	DMD	Lebanese	-
1	Point	IVS59c > G	DMD	Egyptian	-
1	Point Missense	c.7657C > T(p.R2553X)	DMD	Indian	-
1	Point	c.3151 C > T(Arg1051)	DMD	Yemeni	-

We explored potential associations between specific mutation locations and intellectual impairment in our DMD cohort. Among the eleven patients with reported intellectual or learning difficulties, seven patients had deletions or point mutations involving exons associated with the loss of Dp140 or Dp71 isoforms (e.g., exons 44–55 or distal exons >63), which have been implicated in central nervous system involvement. Although the sample size was too small to draw statistically significant conclusions, our findings are consistent with previous reports linking mutations that disrupt brain-expressed dystrophin isoforms to cognitive and behavioral deficits (7). We observed three patients with autism and one with ADHD. These findings underscore the need for neuropsychiatric screening as part of the routine evaluation of DMD patients.

The symptomatic female patient with a *de novo* deletion mutation challenges traditional X-linked inheritance assumptions and emphasizes the need for heightened clinical suspicion in atypical presentations. The BMD patient remained ambulatory and asymptomatic until age 30, reflecting the known milder course of BMD. A particularly rare case of dual pathogenic variants in DMD and LAMA2 highlighted the diagnostic complexity of neuromuscular disorders in consanguineous populations and the value of whole exome sequencing and muscle biopsy for phenotype clarification.

Overall, the outcomes of this review highlights the following: (1) Qatar has one of the earliest age of diagnosis rates in the Middle Eastern region when compared to neighboring countries (9), which reduces missed opportunities to provide a management plan early on before disease progression thus improving patient outcome and increasing life expectancy; (2) Despite early diagnosis there is a low life expectancy of patients in this cohort compared to worldwide data. This can be due cultural attitudes, social stigmas, and healthcare-seeking behaviors that can influence the identification, reporting and early management of DMD and BMD cases. Cultural factors may affect the willingness of individuals and families to seek medical attention, accept management strategies, participate in research studies, or disclose personal health information. Another contributing factor is differences in research priorities, funding availability, and collaboration networks between Qatar and other countries may influence the scope and depth of studies conducted on DMD and BMD. Collaborating with other research centers provides access to resources, expertise, and advanced technologies that may not be readily available in Qatar. By collaborating with leading research centers, our center can accelerate the translation of scientific discoveries into clinical practice. This may involve the development of novel therapeutics, diagnostic tools, or adopting guidelines tailored to the specific needs of DMD and BMD patients in Qatar, ultimately improving the outcomes and quality of life. As well collaboration with international research centers can expand funding opportunities for DMD and BMD research in Qatar by participating in multinational research consortia, accessing grants from global funding agencies, and engaging with philanthropic organizations and importantly ensure the mixed ethnic representation in these studies.

Despite the ambitious attempt in this study, there are some limitations that need to be addressed that include the retrospective nature of the study, relatively small sample size, and incomplete data for some variables. The small number of BMD patients limits the generalizability of findings to this subgroup. Future prospective studies are needed to monitor long-term outcomes and the effectiveness of newer therapies.

## Conclusion

In summary, our study confirms early diagnosis but suboptimal long-term management of dystrophinopathies in Qatar. The findings reveal a need for improved adherence to international care standards, including early initiation and sustained use of corticosteroids, routine cardiac monitoring, and genetic counseling. Multidisciplinary care models must integrate neurology, cardiology, pulmonology, endocrinology, physiotherapy, and neurodevelopmental services. Qatar's healthcare system, with its centralized and well-equipped facilities, is well-positioned to lead regional initiatives such as national DMD/BMD registries, clinical protocols, and access to advanced therapies like exon-skipping and gene therapy. Precision medicine approaches and multidisciplinary frameworks will be critical to optimize care and outcomes for DMD and BMD patients across the region.

## Data Availability

The raw data supporting the conclusions of this article will be made available by the authors, without undue reservation.
